# Acai Berry Extracts Can Mitigate the L-Glutamate-Induced Neurotoxicity Mediated by N-Methyl-D-Aspartate Receptors

**DOI:** 10.3390/brainsci15101073

**Published:** 2025-10-01

**Authors:** Maryam N. ALNasser, Nirmal Malik, Abrar Ahmed, Amy Newman, Ian R. Mellor, Wayne G. Carter

**Affiliations:** 1 Department of Biological Sciences, College of Science, King Faisal University, P.O. Box No. 400, Al-Ahsa 31982, Saudi Arabia; malnasser@kfu.edu.sa; 2 Clinical Toxicology Research Group, School of Medicine, Royal Derby Hospital Centre, University of Nottingham, Derby DE22 3DT, UK; 3 School of Life Sciences, Faculty of Medicine and Health Sciences, University of Nottingham, Nottingham NG7 2RD, UK; amyjennewman@gmail.com (A.N.); ian.mellor@nottingham.ac.uk (I.R.M.); 4 Faculty of Pharmacy, Punjab University College of Pharmacy, University of the Punjab, Mall Road, Lahore 54000, Pakistan; drnirmalmalik9@gmail.com (N.M.); abrar.pharmacy@pu.edu.pk (A.A.)

**Keywords:** acai berry, excitotoxicity, L-glutamate, neuroprotection, oxidative stress, stroke

## Abstract

Background/Objectives: Stroke is the second leading cause of death worldwide. There is an unmet need to manage stroke pathophysiology, including L-glutamate (L-Glu)-mediated neurotoxicity. The acai berry (*Euterpe* sp.) contains phytochemicals with potentially nutraceutical content. The aim of this study was to assess the ability of acai berry extracts to counter L-Glu neurotoxicity using human differentiated TE671 cells. Methods: The cytotoxicity of L-Glu and acai berry extracts was quantified using 3-(4,5-dimethylthiazol-2-yl)-2,5-diphenyltetrazolium bromide (MTT) and lactate dehydrogenase (LDH) assays. Mitochondrial function was examined by a quantitation of cellular ATP levels, the maintenance of the mitochondrial membrane potential (MMP), and the production of reactive oxygen species (ROS). Whole-cell patch-clamp recordings monitored the activation of N-methyl-D-aspartate receptors (NMDARs). Candidate phytochemicals from acai berry extracts were modeled in silico for NMDAR binding. Results: L-Glu significantly reduced cell viability, ATP levels, the MMP, and increased cellular ROS. Generally, acai berry extracts alone were not cytotoxic, although high concentrations were detrimental to ATP production, maintenance of the MMP, and elevated ROS levels. Whole-cell patch-clamp recordings revealed that the combined addition of 300 µM L-Glu and 10 µM glycine activated currents in differentiated TE671 cells, consistent with triggering NMDAR activity. Acai berry extracts ameliorated the L-Glu-induced cytotoxicity, mitochondrial dysfunction, elevated ROS levels, and limited the NMDAR-mediated excitotoxicity (*p* < 0.001–0.0001). Several virtual ligands from acai berry extracts exhibited high-affinity NMDAR binding (arginine, 2,5-dihydroxybenzoic acid, threonine, protocatechuic acid, and histidine) as possible candidate receptor antagonists. Conclusions: Acai berry phytochemicals could be exploited to reduce the L-Glu-induced neurotoxicity often observed in stroke and other neurodegenerative diseases.

## 1. Introduction

Strokes are estimated to afflict 13.7 million people worldwide and result in over 6 million annual fatalities, making them the second most common cause of mortality [1,2]. In the UK, there are over 113,000 people who suffer a stroke annually, with approximately 1 million stroke survivors, although this is likely to increase by 60% per year over the next dozen years due to an aging population and treatment improvements [3]. The socio-economic impact of stroke is extensive with an annual cost for long-term care and patient rehabilitation as well as lost employment costs estimated at GBP 26 billion in the UK [4]. Globally, stroke costs have been estimated at over USD 891 billion [5].

Strokes are neurological diseases caused by a disruption or rupture of the blood vessels of the central nervous system (CNS), which result in a restriction of blood flow to the brain [1]. Strokes are classified into ischemic and hemorrhagic, with the former more common, constituting >80% of strokes [1]. For ischemic strokes, this inadequate blood flow to the brain reduces the supply of nutrients and oxygen necessary to sustain brain function [1]. Hemorrhagic strokes (10% to 20% of all strokes) occur when blood leaks out of vessels into the skull or surrounding tissues [1,6]. By either means, a stroke results in an infarct of neuronal tissue [6,7]. The gradual or sudden hypoperfusion of blood to the brain initiates an ischemic cascade with a diminished cellular energy supply. There follows an elevation of extracellular glutamate (L-Glu) and intracellular calcium [Ca^2+^]_i_ increase and overload (excitotoxicity). Oxidative and/or nitrosative stress is triggered as well as a disruption to ion homeostasis, blood–brain barrier (BBB) damage, cytotoxic and vasogenic oedema, peri-infarct depolarization, and neuroinflammation. Collectively, this drives cell death through the apoptotic and necrotic death of the cells of the neurovascular unit (neurons, astrocytes, pericytes, and vascular endothelial cells) [1,8].

An intravenous thrombolysis is used to treat the ischemic stroke, in an attempt to restore rapid reperfusion, and interrupt the pathophysiology of ischemic disease [9,10]. However, the current treatment options are limited. They can assist reperfusion (such as by administration of a tissue plasminogen activator (t-PA), for example, alteplase) [10,11], but cannot completely block all of the pathological events.

One of the major pathological mechanisms in stroke is the initiation of excitotoxicity. This is a contributor to cerebral post-ischemic stroke damage, and a pathomolecular process that involves the production of excessive L-Glu and prolonged activation of *N*-methyl-D-aspartate receptors (NMDARs) [1,8,12,13,14,15,16]. The post-stroke elevation of L-Glu triggers several pathological cascades as well as excitotoxicity, including oxidative/nitrosative stress, mitochondrial malfunction, and ionic imbalance in the ischemic brain tissue, which collectively result in extensive brain damage [1,8,12,13,14,15,16]. Clinical trials have been undertaken to test agents able to reduce the L-Glu excitotoxicity in stroke patients. These act through either inhibition of L-Glu release, antagonism of the NMDARs, and/or inhibition of death-signaling cascades downstream of the NMDAR [14,15,16].

Collectively, treatments for ischaemic stroke have focused on neuronal protection and regeneration mechanisms to restore brain functional deficits [17]. Although there is not yet a Food and Drug Administration (FDA)-approved neuroprotective treatment, there are a range of modalities currently being tested, including antioxidants and free radical scavengers, and anti-inflammatories [18].

The acai berry, a fruit indigenous to the Caribbean islands and the Amazon region of South America, is produced by tropical palm trees belonging to the *Euterpe* genus [19]. An array of pharmacological effects, including anti-inflammatory, antioxidant, anti-carcinogenic, and neuroprotective activities have been attributed to the bioactive constituents of acai berries [19,20,21,22,23,24].

Acai berry extracts provide neuroprotective effects through their antioxidant and anti-inflammatory properties, ability to inhibit toxic protein aggregation, and the restoration of mitochondrial function and Ca^2+^ homeostasis [19]. Accordingly, acai berry phytochemicals may be able to counteract multiple components of stroke pathology. Neuron death after stroke onset is associated with L-Glu-mediated excitotoxicity, so the discovery of agents that inhibit or moderate L-Glu toxicity may help to treat strokes [14]. Hence, the administration of antioxidants, or L-Glu and NMDAR antagonists, could provide neuronal protection or/and assist with tissue regeneration [1,25,26].

Previously, we showed that acai berry extracts are neuroprotective when co-applied with L-Glu to an undifferentiated human neuroblastoma (SH-SY5Y) cell line, as well as pluripotent ReNCell CX cells [27]. In this research study, we have extended this work, with a study aim to examine the neuroprotective capabilities of acai berry extracts to counter L-Glu neurotoxicity in differentiated human rhabdomyosarcoma TE671 cells, which have demonstrable NMDAR presence and activity [28,29,30,31]. Hence, a study objective was to specifically examine the impact of acai berry extracts on L-Glu excitotoxicity using whole-cell patch-clamp recordings. Furthermore, from an analysis of known phytochemicals in acai berry extracts, we used virtual screening to consider candidate compounds with relatively high-affinity binding to the NMDAR, which could provide NMDAR antagonism.

## 2. Materials and Methods

### 2.1. Chemicals and Reagents

Chemicals and media were purchased from Sigma-Aldrich (Poole, UK), unless stated otherwise.

### 2.2. Human Rhabdomyosarcoma TE671 Cell Culture and Differentiation

TE671 cells were purchased from the European Collection of Authenticated Cell Cultures (ECACC, catalogue no. 89071904). Cells were cultured and differentiated as described previously [27]. Briefly, Dulbecco’s modified Eagle’s medium (DMEM; D6546, Sigma-Aldrich, Poole, UK) with 10% (*v*/*v*) fetal bovine serum (FBS), 2 mM glutamine, and 1% penicillin–streptomycin solution was used to cultivate the TE671 cells at 37 °C under humidified conditions of 5% CO_2_ in 95% air. Cellular differentiation was initiated by the application of 400 µM N6,2′-O-dibutyrylladenosine 3′,5′-cyclic monophosphate sodium salt (dbcAMP) in serum-free DMEM containing 2 mM L-glutamine, and 1% Penicillin/Streptomycin. Differentiated TE671 cells (dTE671) displayed long and branched neuritic arborization, consistent with a neuronal phenotype (refer to Supplementary Figure S1) [32].

### 2.3. Preparation of L-Glutamic Acid (L-Glu), Acai Berry Aqueous and Ethanolic Extracts and Cell Treatments

The source of the acai berry was a standardized commercially available freeze-dried acai pulp and skin powder, purchased from NaturaleBio (Organic product under EU Directive 834/2007, purchased via Amazon.co.uk) [33]. Aqueous and ethanolic extracts of the acai berry and stocks of L-Glu were prepared as detailed previously [27]. After cells were differentiated using dbcAMP, L-Glu neurotoxicity was assessed by incubation with L-Glu over a broad concentration range of 0.137–100 mM for 24 or 48 h. The differentiated TE671 cells were treated with acai berry extracts (0.001 µg/mL–1000 µg/mL or 0.001 µg/mL–10 µg/mL) for 24 or 48 h. To investigate the neuroprotective capabilities of the acai berry extract, it was combined with L-Glu at concentrations of 11 mM or 100 mM.

### 2.4. 3-(4,5-Dimethylthiazol-2-yl)-2,5-Diphenyltetrazolium Bromide (MTT) Assay

An MTT assay evaluated cell viability of differentiated TE671 cells in response to L-Glu, acai extracts, and a coincubation with both agents. TE671 cells were plated in 96-well plates (1 × 10^4 ^cells/well) and after differentiation (using dbcAMP) treated with the compounds at the concentrations and durations described above. After treatment, MTT (0.5 mg/mL) was applied to each well, as previously described [27]. After 2 h, the formazan crystals that were formed were dissolved in dimethyl sulfoxide (DMSO), and then the absorbance was measured at 570 nm using a VarioskanTM LUX multimode microplate reader (ThermoFisher Scientific, Waltham, MA, USA). The absorbance of control wells (absence of test compounds) was set at 100% cell viability, and DMSO was used as a vehicle control.

### 2.5. Lactate Dehydrogenase (LDH) Assay

Quantitation of the levels of LDH released into the culture media as a consequence of cell membrane rupture were used to confirm changes in cell viability in response to treatments with L-Glu, acai berry extracts, or a combination of both agents. Cells were seeded in 96-well plates (1 × 10^4^ cells/well), differentiated with dbcAMP, and then treated with L-Glu, acai berry extracts, or both as described above. LDH released into the medium was quantified using an LDH cytotoxicity test kit (ThermoFisher Scientific, Waltham, MA, USA) according to a previous publication [27]. Spectrophotometric measurements were recorded at 490 nm and 680 nm in a VarioskanTM LUX multimode microplate reader (ThermoFisher Scientific, Waltham, MA, USA).

### 2.6. Adenosine 5′-Triphosphate (ATP) Assay

A bioluminescence assay was used to quantify intracellular ATP levels as an alternative method to assess a cellular toxicity response and a decline in cell viability [34,35]. As a result of cellular injury, there is a reduction in ATP synthesis and the rapid release of endogenous ATPase depletes any remaining ATP [35,36]. TE671 cells were plated in 6-well plates (2 × 10^5 ^cells/well), differentiated with dbcAMP, and then treated with L-Glu or acai berry extract or both, as described above. Cells were homogenized and the levels of ATP quantified using an ATP Bioluminescence Assay Kit, CLS II (Sigma-Aldrich, Poole, UK) according to the manufacturer’s protocol [27].

### 2.7. Measurements of Mitochondrial Membrane Potential (MMP)

The integrity of mitochondria after cellular treatments was assessed by quantitation of the mitochondrial membrane potential (MMP). Cells were grown in 96-well plates (1 × 10^4 ^cells/well) and after differentiation using dbcAMP, cells were treated with L-Glu, acai berry extracts, or both as described above. Cells were stained by the application of 50 nM MitoTracker^®^ Green FM (ThermoFisher Scientific, Waltham, MA, USA) into live cells for 30 min at 37 °C, as detailed previously [27]. After removing the stain, fresh PBS was added, and the fluorescence was measured using excitation at 490 nm and emission at 516 nm using a VarioskanTM LUX multimode microplate reader (ThermoFisher Scientific, Waltham, MA, USA). Negative controls were untreated cells labeled with 50 nM Mito-Tracker^®^ Green FM, and carbonyl cyanide-4-(trifluoromethoxy) phenylhydrazone (FCCP)-treated cells were used as a positive control for mitochondrial membrane uncoupling.

### 2.8. 2,7-Dichlorodihydrofluorescein Diacetate Assay

Levels of reactive oxygen species (ROS) generated in cells were quantified using a fluorogenic 2,7-dichlorodihydrofluorescein diacetate (DCFHDA) assay based on published protocols [27,37,38]. Cells were grown in 96-well plates (1 × 10^4 ^cells/well) and differentiated using dbcAMP and then exposed to L-Glu, acai berry extracts, or both, as described above, along with 50 μM of the non-fluorescent dye DCFHDA, for 3 or 6 h at 37 °C. As a negative control, cells were stained just with 50 μM DCFHDA, and blanks were untreated cells. Cells incubated with 500 μM H_2_O_2_ for 30 min were used as a positive control cellular stressor for ROS production. Following a wash with PBS, fresh PBS was added to the treated cells and the fluorescence signal from oxidized DCFHDA measured at 485 nm excitation and 535 nm emission using a VarioskanTM LUX multimode microplate reader (ThermoFisher, Waltham, MA, USA).

### 2.9. Whole-Cell Patch-Clamp Assay

Whole-cell patch-clamp electrophysiology was performed on differentiated TE671 cells using an Axopatch 200A patch-clamp amplifier (Axon Instruments, Union City, CA, USA) at a holding potential of −50 mV as previously described [27]. Cells were grown and differentiated on glass coverslips (5 × 18 mm) for transfer to a perfusion bath mounted on the stage of an inverted microscope, and perfused continuously with Mammalian Ringer solution at 5 mL/min, according to a previous publication [27]. Test chemicals were as follows: 300 µM L-Glu + 10 μM Gly; 300 µM L-Glu + 10 µM Gly + 1 µM dizocilpine (MK-801); 300 µM L-Glu + 10 μM Gly + 1 mM Mg^2+^; 300 µM L-Glu + 10 μM Gly + acai aqueous extract (at a concentration of 0.001, 1, or 1000 µg/mL). Twenty differentiated TE671 cells were examined in each assay.

### 2.10. In Silico Studies

#### 2.10.1. Molecular Docking of Acai Berry Extract Compounds to NMDA Receptors

Molecular docking analysis for compounds from acai berry extracts [27] (Supplementary Table S1) was performed using the Schrödinger suite, version 13.2, LLC, 2022.2 (New York, NY, USA). The identified compounds were docked in the groove of the binding site in 5H8F, which is the crystal structure of the GluN1/GluN2A ligand binding domain (LBD) with a resolution of 2.97 Å. For in silico modeling studies, the NMDA receptor complex structure was used from the cryo-electron microscopy image (accession ID: 5H8F) available at the RCSB-Protein Data Bank (PDB) (https://www.rcsb.org/structure/5H8F, accessed on 27 July 2025). The target protein’s three-dimensional structure (NMDA receptor with PDB ID: 5H8F) is shown in Supplementary Figure S2.

Protein processing of the acquired PDB structure was undertaken to provide the parent protein structure with chemical correctness using The Protein Preparation Wizard in the Maestro v13.0 Schrödinger, LLC., 2023.1 software package (https://my.schrodinger.com/download/software/, accessed on 27 July 2025). An optimized protein structure is required in order to use the molecular docking tool Glide (Grid-based Ligand Docking with Energetics by Schrödinger, LLC, 2023.1 software package) in order to generate accurate docking calculations. The Protein Preparation Wizard was used to eliminate the co-crystallized ligand and the associated ions and water molecules. Missing residues were added, chosen chains were given polar hydrogens to satisfy their valences, formal charges were changed, and appropriate bond orders were assigned. After protonating the structure to a pH of 7.0, the structure was reduced using the Optimized Potentials for Liquid Simulations (OPLS2005) force field. This resulted in an optimal configuration of the protein structure suitable for molecular docking experiments.

#### 2.10.2. Ligand Selection and Preparation

To consider their neuroprotective properties, known phytochemicals present within acai berry extracts [27] were systematically evaluated as putative NMDAR ligands. The 2D sketcher software tool, available in Maestro v13.0 Schrödinger, LLC., 2023.1 (https://my.schrodinger.com/download/software/, accessed on 27 July 2025) was used to draw the structures of the possible NMDAR ligands, with ligands structurally adjusted for precise docking results using Lig-Prep, available in Maestro v13.0 Schrödinger, LLC., 2023.1 (https://my.schrodinger.com/download/software/, accessed on 27 July 2025). Lig-Prep conducted a variety of tasks, including the chirality correction for all input structures, ring conformation optimization, and correction of bond order and bond length. Undesired structures were removed, and the built-in Epik module was used to produce probable ionization states for each ligand at a pH of 7. The OPLS-2005 force field was then used to minimize the energy of the ligands in question, and these low-energy 3D optimized ligand structures were employed for molecular docking.

#### 2.10.3. Molecular Docking

The potential binding site to the NMDAR was predicted for each ligand after structural refinement. The ligand glutamate was removed from the binding pocket of the target protein during protein preparation and then with the aid of the Glide Receptor Grid Generation panel which exposed the x, y, z coordinates, a grid was generated at the active site of the receptor. Thereafter, docking calculations were performed with Glide Extra Precision (XP-visualizer module) within the defined receptor grid. Each ligand was constructed in several spatial directions relative to the NMDAR active site with the use of flexible docking algorithms. Subsequently, XPGlide performed heuristic high-throughput virtual screening (HTVS) and removed unproductive ligand poses that had high unfavorable energies, predicting the best energies for conformation poses for each ligand. The selected ligand poses were then reduced using the OPLS-AA (All-Atom Optimized Potentials for Liquid Simulations) forcefield, and the best-bound energy minimized ligand poses were ranked based on their binding affinities with the NMDAR and docking score. The ligand conformations with higher and comparative negative docking scores with L-glutamate, which was utilized as the reference ligand, were then collated in rank order and examined utilizing molecular mechanics/generalized Born surface area (MM/GBSA) studies.

#### 2.10.4. Molecular Mechanics/Generalized Born Surface Area (MM/GBSA) Simulations

The Prime module of the Maestro v13.0 Schrödinger, LLC, 2022.1 (https://my.schrodinger.com/download/software/, accessed on 27 July 2025) software package was utilized for MM/GBSA analysis to predict the binding free energies (∆Gbind) of the top scoring ligands from the docking study, as described [39]. The docked complexes were minimized initially with the OPLS-2005 force field, which also calculates the binding free energies of the system’s optimized endpoints: free target protein, free ligand, and protein–ligand interaction complex.

#### 2.10.5. Molecular Dynamics (MD) Simulations

The ligands with the strongest interaction with the NMDAR were further assessed for interaction stability through MD simulation studies using Desmond-Maestro v13.0 Schrödinger, LLC., 2022. Default settings were utilized due to the software’s high-performance algorithms. To simulate the aquatic environment, an equilibrated orthorhombic water box centered on the mass center of each ligand was employed to solve the docked complex. To electrically neutralize the system before running simulations, Na^+^ and Cl^−^ ions were used. The generated system was then reduced with a limited-memory Broyden–Fletcher–Goldfarb–Shanno (LBFGS) method and equilibrated to match the applied force field, OPLS3e. The applied force field compensated for the system’s component interacting forces, with simulations run for 200 ns using an NPT (Normal Temperature and Pressure) ensemble at 300 K and 1.01325 bar pressure. Following that, a statistical examination of the system’s particles’ velocities, energies, and coordinates was used to assess how well a ligand binds to the target’s active site. Root mean square deviation (RMSD) analysis was used to determine the backbone amino acids implicated in the docking complex’s binding and consequently its stability, as described previously [40].

### 2.11. Statistical Analysis

The results from control versus treatment group assays were calculated and displayed as means ± standard error of the mean (SEM). The concentration of L-Glu that produced 50% inhibition (IC_50_) was determined by non-linear regression analysis. One-way ANOVA tests with Tukey’s or Dunnett’s multiples comparisons post-tests were used to compare between groups. A *p*-value of <0.05 was considered statistically significant. All statistical tests were performed using PRISM v9 (GraphPad Software Inc., San Diego, CA, USA).

## 3. Results

### 3.1. L-Glu and High Acai Berry Concentrations Reduce the Viability of dTE671 Cells

**Figure 1:**
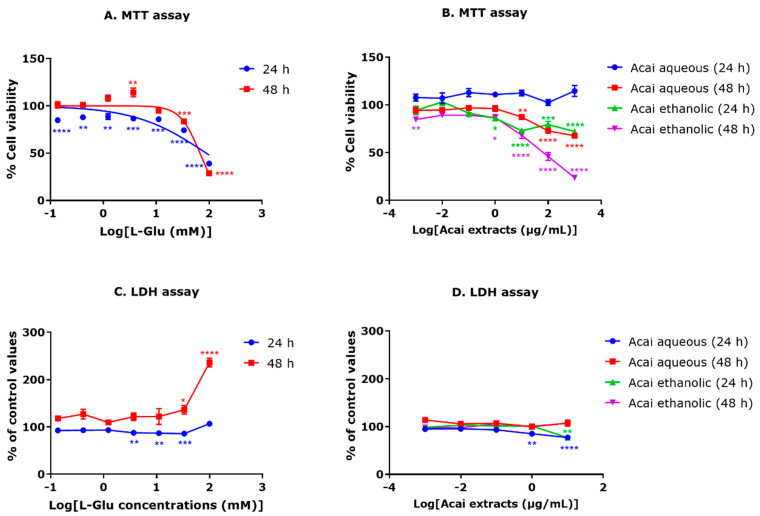
An assessment of dTE671 cell viability in response to L-Glu and acai berry extracts. L-Glu or acai berry extracts were applied to dTE671 cells for 24 or 48 h and cytotoxicity was assessed using an MTT (**A**,**B**) or LDH assay (**C**,**D**). Plotted data points are means ± SEM from three independent experiments (n = 3). Differences between means were evaluated using a one-way ANOVA followed by Dunnett’s multiple comparisons post-test. For marked statistical significance: * *p* < 0.05; ** *p* < 0.01; *** *p* < 0.001; and **** *p* < 0.0001, in comparison to vehicle controls.

Differentiated TE671 cells were treated with L-Glu and the effect on cell viability was quantified using an MTT assay. After a 24 h exposure to concentrations of 0.137–100 mM of L-Glu, there was a significant reduction in cell viability by approximately 10–25% (Figure 1A). Incubation for 48 h with L-Glu reduced cell viability at concentrations of 33.33 and 100 mM (17% reduction, *p* = 0.0008, and 72% reduction, *p* < 0.0001, respectively). A concentration of 3.7 mM L-Glu for 48 h also induced an anomalous increase in MTT readings (14% increase, *p* = 0.0047) (Figure 1A). The estimated IC_50_ values from a non-linear regression for L-Glu toxicity to dTE671 cells were 88.01 mM after 24 h and 67.03 mM after 48 h. The cytotoxicity of acai berry extracts to dTE671 cells was also assessed using the MTT method. After a 24 h exposure to acai berry aqueous extracts for concentrations of 0.001–1000 µg/mL, there was an increased cell metabolic activity (MTT optical density readings) of about 2–14% compared to controls, but this did not reach statistical significance (Figure 1B). After a 48 h incubation with the aqueous acai berry extract at concentrations of 0.001–1 µg/mL, there was a slight decrease in cell viability, but this was also not significant. By contrast, cellular exposure for 48 h to the aqueous acai berry extract at the higher concentrations of 10–1000 µg/mL resulted in a significant reduction in cell viability by 13–33% (*p* < 0.001–*p* < 0.0001) (Figure 1B). The acai ethanolic extract was more toxic to cells and reduced cell viability after 24 h (14–28% decrease) and 48 h (14–77% decrease) at the higher concentration tested (1–1000 µg/mL). There was also an anomalous reduction in cell viability after a 48 h exposure to 0.001 µg/mL of the ethanolic extract (16% (*p* = 0.005) (Figure 1B).

In order to provide an independent method for the assessment of the effect of L-Glu on cell viability, L-Glu neurotoxicity to dTE671 cells was assessed using the formation of extracellular LDH (Figure 1C). A 24 h application of L-Glu to dTE671 cells did not induce observable toxicity over the concentration range of 0.137–100 mM, with an increased release of extracellular LDH only observed at 100 mM, and this was not statistically significant (Figure 1C). Furthermore, the levels of LDH activity in medium from cells exposed to 3.7–33.33 mM L-Glu for 24 h were slightly reduced compared to control cell exposures. After a 48 h exposure to L-Glu, LDH production increased in an approximately concentration-dependent manner that reached statistical significance at L-Glu concentrations of 33.33 and 100 mM (increased by 36% and 136%, respectively) (Figure 1C). LDH assays indicated that acai berry extracts were not generally toxic to dTE671 cells, with minor reductions in the LDH produced after 24 h incubations with 1 and 10 µg/mL of the aqueous extract and 10 µg/mL of the ethanolic extract (Figure 1D).

### 3.2. Acai Berry Extracts Protect dTE671 Cells from the L-Glu-Induced Loss of Cell Viability

**Figure 2:**
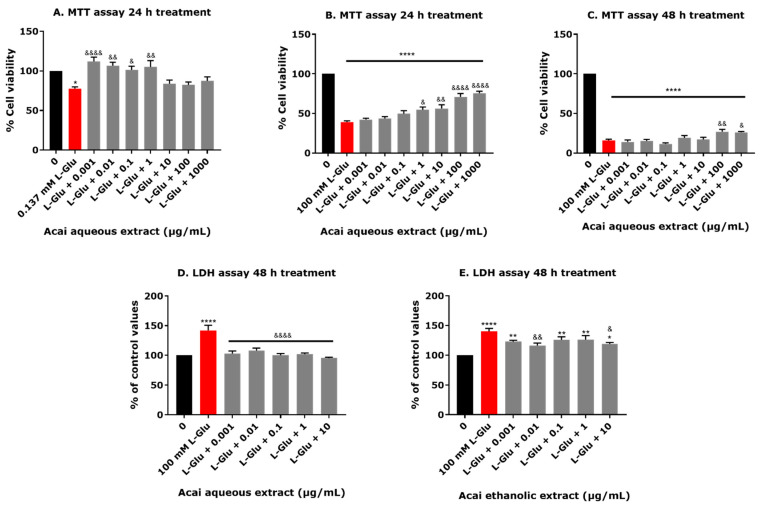
An assessment of dTE671 cell viability in response to coincubation of L-Glu with acai berry extracts. The cytotoxicity of 0.137 or 100 mM L-Glu alone (red columns) or from co-incubations with acai berry extracts (grey columns) was determined after 24 or 48 h using MTT (**A**–**C**) or LDH assays (**D**,**E**). Data points are presented as means ± SEM from three independent experiments (n = 3 per experiment) with optical density values normalized to the mean of the negative control and expressed as a percentage. Differences between means were evaluated using a one-way ANOVA followed by Dunnett’s multiple comparisons post-test. For marked statistical significance in comparison to a control in the absence of L-Glu: * *p* < 0.05; ** *p* < 0.01; **** *p* < 0.0001. For marked significance compared with the 0.137 or 100 mM L-Glu treatments: &, *p* < 0.05; &&, *p* < 0.01; &&&&, *p* < 0.0001.

The neuroprotective capability of the aqueous extract from acai berries was examined after a cellular challenge with 0.137 or 100 mM L-Glu for 24 or 48 h in dTE671 cells. Cell viability was quantified using an MTT assay. In response to 0.137 mM L-Glu alone, there was a 22.5% lowering of cell viability after 24 h, but the coincubation with all concentrations of acai berry aqueous extract improved cell survival by 5–34% for concentrations of 0.001–1 µg/mL (*p* = 0.0197–*p* < 0.0001) (Figure 1A). Upon incubation with 100 mM L-Glu for 24 h, only approximately 40% of the cells remained viable, which was significantly improved in a concentration-dependent manner (by approximately 15–36%) by the co-application of acai berry aqueous extract at concentrations of 1–1000 µg/mL (Figure 2B). Aqueous acai berry extract at 100 μg/mL and 1000 μg/mL significantly improved cell viability after a 48 h incubation with 100 mM L-Glu, with an approximate 11% increase in viability over L-Glu alone (Figure 2C). An independent assessment of cell viability using an LDH assay revealed that the application of 100 mM L-Glu for 48 h increased extracellular LDH activity by 42%, and this was significantly reduced by about 35–51% (*p* < 0.0001) for co-incubations of L-Glu with the acai berry aqueous extract (Figure 2D). Similarly, the toxicity triggered from exposure to 100 mM L-Glu for 48 h (40% increase in extracellular LDH activity) was inhibited by approximately 16–26% with the acai ethanolic extract co-application, with significant effects at concentrations of 0.001–10 µg/mL (Figure 2E).

### 3.3. Acai Berry Extracts Countered the L-Glu-Induced Reduction in ATP Levels in dTE671 Cells

**Figure 3:**
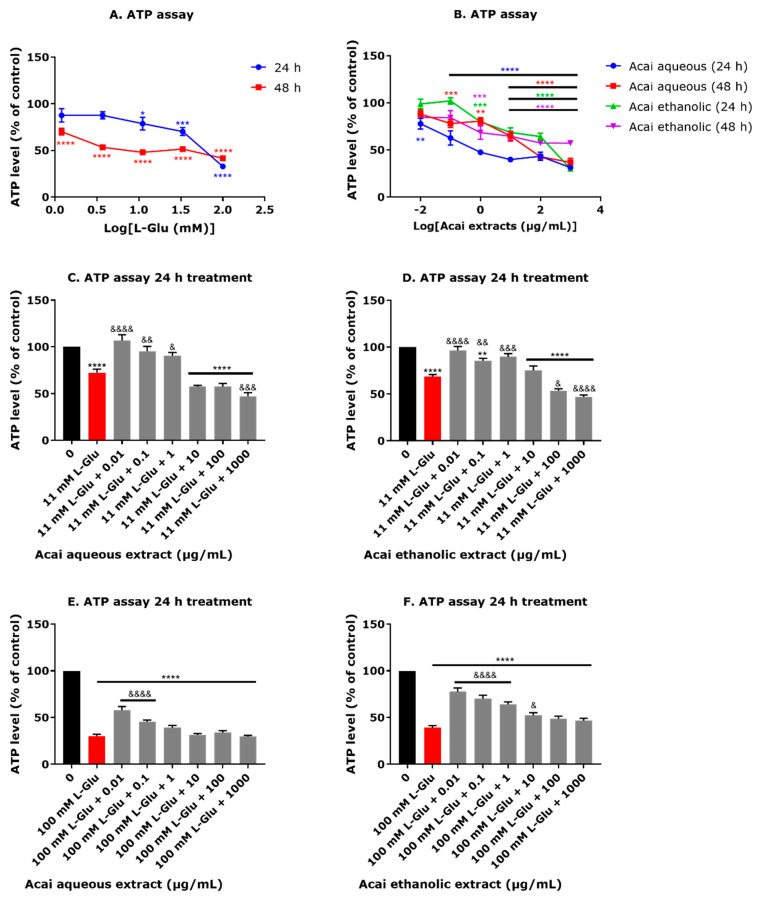
Effects of L-Glu and acai berry extracts on cellular ATP levels in dTE671 cells. ATP levels were quantified in dTE671 cells exposed to L-Glu (**A**) or acai berry extracts (**B**) for 24 or 48 h or after exposure to 11 or 100 mM L-Glu alone (red columns) and different concentrations of acai berry extracts (grey columns) (**C**–**F**) for 24 h. An ATP level percentage relative to the vehicle control was calculated by normalizing the values to the negative control. Data points are presented as means ± SEM from three independent experiments (n = 3 per experiment). Differences between means were evaluated using a one-way ANOVA with Dunnett’s (**A**,**B**) or Tukey’s (**C**–**F**) multiple comparisons post-test. For marked statistical significance in comparison to a control in the absence of L-Glu or acai berry extracts: * *p* < 0.05; ** *p* < 0.01; *** *p* < 0.001; **** *p* < 0.0001. For marked significance in comparison with the L-Glu group: &, *p* < 0.05; &&, *p* < 0.01; &&&, *p* < 0.001; &&&&, *p* < 0.0001.

The application of L-Glu to dTE671 cells induced a concentration-dependent decrease in ATP levels after a 24 or 48 h incubation (Figure 3A). At concentrations of 11.1, 33.3, and 100 mM L-Glu, there were significantly decreased levels of ATP of 22, 30, and 68%, respectively, after 24 h (Figure 3A), giving an estimated IC_50_ of 58.17 mM. Similarly, the incubation of dTE671 cells with L-Glu (over the concentration range of 0.137–100 mM) for 48 h also induced significant reductions in ATP levels (30–60%), and with an estimated IC_50_ of 18.79 mM (Figure 3A). Acai berry extracts applied to the dTE671 cells over the concentration range of 0.01 to 1000 µg/mL induced a concentration-dependent reduction in ATP levels after 24 and 48 h (Figure 3B). There was a 23–69% decline in cellular ATP observed for 24 h incubations with the acai berry aqueous extract and a 22–64% reduction in ATP after 48 h (Figure 3B). Likewise, acai berry ethanolic extracts induced declines of ATP by 21–70% after 24 h and 32–43% after 48 h (Figure 3B). The co-exposure of acai berry extracts with 11 or 100 mM L-Glu provided some neuroprotection and was able to ameliorate the L-Glu-induced decline in ATP levels when the extracts were applied at relatively low concentrations (0.01–1 µg/mL) (Figure 3C–F). Similarly, the co-incubation of L-Glu with these concentrations of the acai berry ethanolic extracts resulted in a maintenance of ATP levels (by approximately 20–30%) when compared with the reduction induced by 11 mM L-Glu alone (Figure 3C,D). Acai berry aqueous extracts at concentrations of 0.01 and 0.1 µg/mL also significantly improved the ATP levels depleted in response to 100 mM L-Glu (27% and 15% increases, respectively) (Figure 3E), whereas the ethanolic extracts significantly improved the ATP levels over the concentration range of 0.01–10 µg/mL (by 38–13%, respectively) from 100 mM L-Glu alone (Figure 3F). Both aqueous and ethanolic acai berry extracts produced concentration-dependent effects on mitigating the L-Glu-induced decline in ATP levels that were highest at the lowest extract concentrations, consistent with their potential for toxicity at higher extract concentrations (Figure 3E,F).

### 3.4. Acai Berry Extracts Countered the L-Glu-Induced Reduction in the MMP in dTE671 Cells

**Figure 4:**
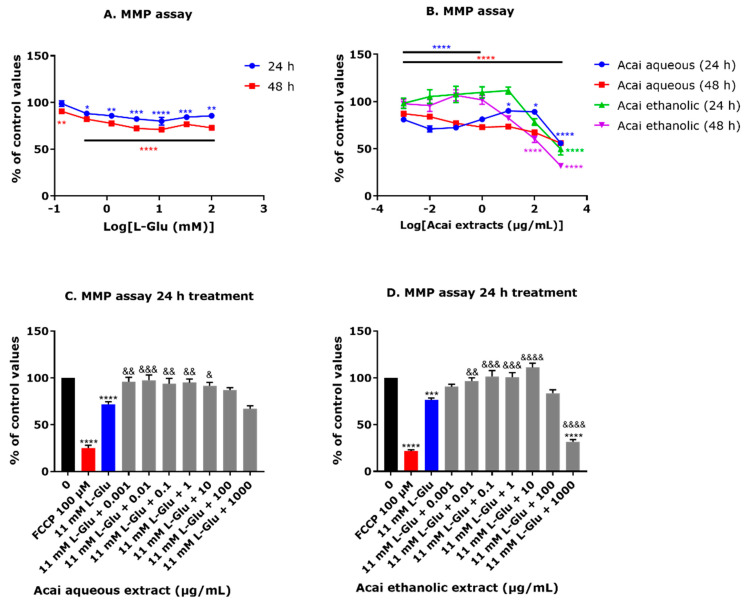
Effects of L-Glu and acai berry extracts on the mitochondrial membrane potential in dTE671 cells. The mitochondrial membrane potential (MMP) level was quantified after exposure to L-Glu (**A**) or acai berry extracts (**B**) for 24 and 48 h, or 11 mM L-Glu alone (blue columns) or 11 mM L-Glu co-incubated with acai berry aqueous (**C**) or ethanolic (**D**) extracts (grey columns) for 24 h. A 24 h incubation with 100 µM carbonyl cyanide-4-(trifluoromethoxy) phenylhydrazone (FCCP) was used as a positive control for MMP uncoupling (red columns). Data points are presented as means ± SEM from three independent experiments (n = 3 per experiment) with fluorescent measurements normalized to the mean of the negative control and expressed as a percentage. Differences between means were evaluated using a one-way ANOVA with Dunnett’s (**A**,**B**) or Tukey’s (**C**,**D**) multiple comparisons post-test. For marked statistical significance in comparison to a control in the absence of L-Glu or acai berry extracts: * *p* < 0.05; ** *p* < 0.01; *** *p* < 0.001; **** *p* < 0.0001. For marked significance in comparison with the L-Glu group: &, *p* < 0.05; &&, *p* < 0.01; &&&, *p* < 0.001; &&&&. *p* < 0.0001.

Exposure to L-Glu at concentrations in the range of 0.137–100 mM significantly decreased the MMP level in dTE671 cells by approximately 12–20% after 24 h and 10–30% after 48 h (Figure 4A). Acai berry aqueous extract at concentrations of 0.001–1000 µg/mL also significantly reduced the MMP level by 10–45% after 24 h and 13–45% after 48 h. Furthermore, incubation with the higher concentrations of acai berry aqueous or ethanolic extracts (100 and 1000 µg/mL) resulted in a significant decline in the MMP after 24 h by 10 and 45%, and 21 and 51%, respectively, and after 48 h by 32 and 45%, and 40 and 69%, respectively (Figure 4B). The co-incubation of acai berry aqueous and ethanolic extracts with 11 mM L-Glu for 24 h ameliorated the L-Glu-induced reduction in the MMP (Figure 4C,D). Acai aqueous extract at concentrations of 0.001–10 µg/mL significantly restored the MMP level by 20–26% (Figure 4C). However, co-incubation of L-Glu (11 mM) with the higher acai berry aqueous concentrations of 100 and 1000 µg/mL did not restore the MMP and for the latter, induced a further drop in the MMP than that from L-Glu alone, consistent with acai berry extract toxicity at relatively high concentrations (Figure 4C). Similarly, the co-incubation of acai berry ethanolic extract (0.01–10 µg/mL) with L-Glu (11 mM) showed a significant concentration-dependent increase in the MMP level (by approximately 20–35%) (Figure 4D), and likewise, the ethanolic extract (1000 µg/mL) failed to protect from L-Glu toxicity and induced a more extensive reduction in the MMP (Figure 4D).

### 3.5. Acai Berry Extracts Countered the L-Glu-Induced Production of Reactive Oxygen Species in dTE671 Cells

**Figure 5:**
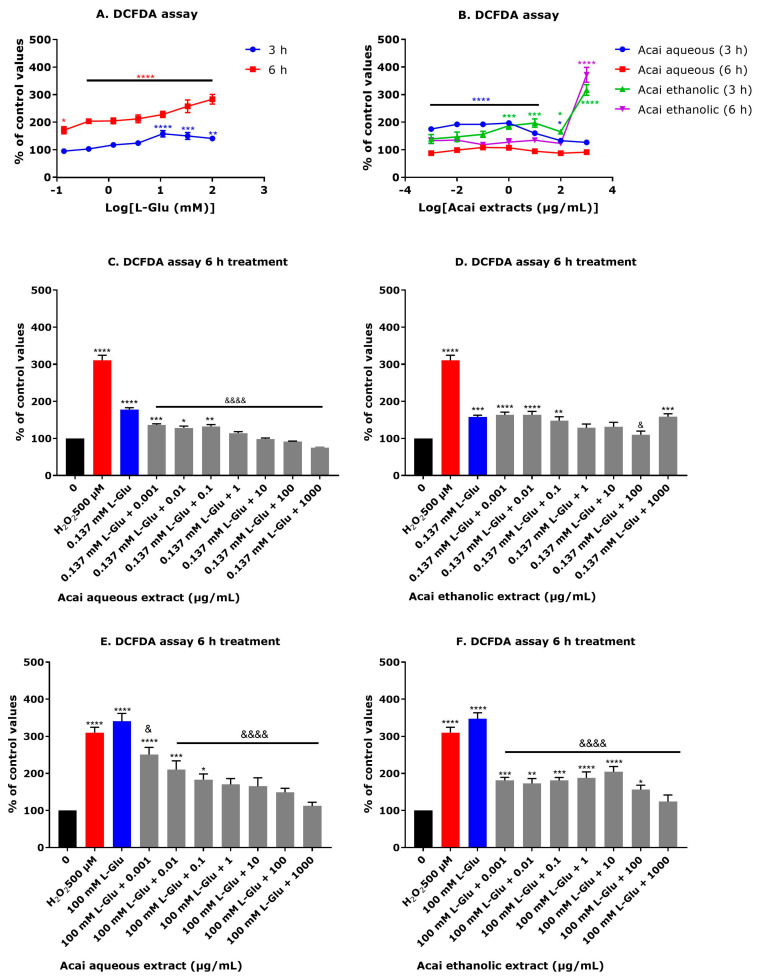
Effects of L-Glu and acai berry extract on the levels of cellular reactive oxygen species in dTE671 cells. The levels of cellular reactive oxygen species (ROS) were quantified using a DCFDA fluorescence assay in response to incubation of dTE671 cells with L-Glu (**A**), and acai berry extracts (**B**) for 3 and 6 h, and after exposure to 0.137 and 100 mM L-Glu (blue columns) and concentrations (0.001–1000 µg/mL) of acai berry aqueous (**C**,**E**) and ethanolic (**D**,**F**) extracts (grey columns) for 6 h. Hydrogen peroxide (H_2_O_2_) at 500 µM for 30 min was used as a positive control (red columns) for the generation of ROS. Data points are presented as means ± SEM from three independent experiments (n = 3 per experiment) and were normalized to the mean of the negative control and expressed as a percentage. Differences between means were evaluated using a one-way ANOVA with Dunnett’s (**A**,**B**) or Tukey’s (**C**–**F**) multiple comparisons post-test. For marked statistical significance in comparison to a control in the absence of L-Glu or acai berry extracts: * *p* < 0.05; ** *p* < 0.01; *** *p* < 0.001; **** *p* < 0.0001. For marked significance in comparison with the L-Glu group: &, *p* < 0.05; &&&&. *p* < 0.0001.

The application of L-Glu (0.137–100 mM) to dTE671 cells induced an increase in reactive oxygen species (ROS) after 3 and 6 h that was concentration dependent (Figure 5A). A 3 h incubation with L-Glu significantly increased ROS production by approximately 40–58% at concentrations of 11.1–100 mM and after a 6 h incubation, all L-Glu concentrations (0.137–100 mM) caused significantly increased ROS levels (70–183%). The incubation of dTE671 cells with acai berry extracts alone also induced some ROS production (Figure 5B). A 3 h incubation of acai berry aqueous extract at concentrations of 0.001–10 µg/mL significantly increased ROS levels by 60–96%, but this extract was without effect at 6 h. The ethanolic extract of acai berries at concentrations of 1–1000 g/mL increased ROS levels by 65–216% above controls after 3 h, but only the 1000 µg/mL concentration significantly increased ROS levels after 6 h by 271% above controls (Figure 5B). The coincubation of acai berry extracts with L-Glu resulted in a reduction in the levels of induced ROS (Figure 5C–F). After a 6 h exposure to 0.137 mM L-Glu and coincubation with the acai berry aqueous extract (0.001 and 10 µg/mL), there was a concentration-dependent reduction in ROS levels (40–90%) (Figure 5C). Likewise, the coincubation of acai berry ethanolic extracts (0.1–100 µg/mL) reduced the ROS levels induced with 0.137 mM L-Glu, and this was predominantly concentration-dependent and significant at 100 µg/mL (48% decrease) (Figure 5D). The levels of ROS produced in response to 100 mM L-Glu for 6 h were also significantly reduced (89–228%) and in a concentration-dependent fashion, for coincubations with the acai berry aqueous extract and L-Glu (Figure 5E). Similarly, the acai berry ethanolic extracts (0.001–1000 µg/mL) significantly decreased the ROS levels (by 166–223%) that were triggered by an incubation with 100 mM L-Glu (Figure 5F).

### 3.6. Acai Berry Extracts Inhibited L-Glu and Gly-Activated Currents in dTE671 Cells

**Figure 6:**
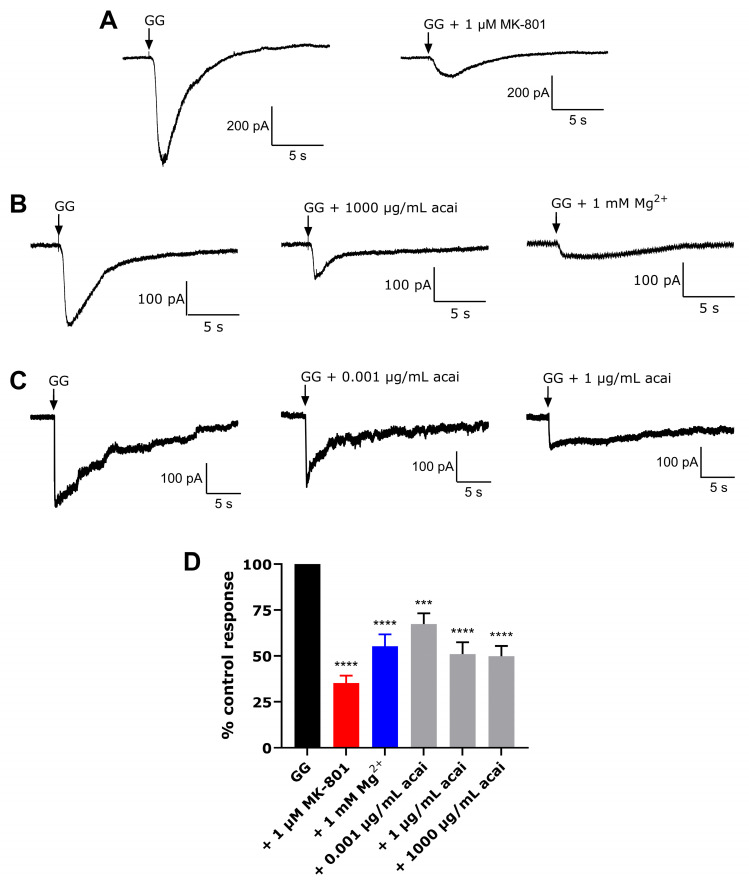
Effects of iGluR agonists, NMDAR antagonists, and acai berry extracts on dTE671 cells using whole-cell patch clamp recordings. The inward current in dTE671 cells at a holding potential of −50 mV was measured in response to 300 µM L-Glu + 10 µM Gly (GG), and GG + 1 μM MK-801 (**A**); GG, GG + 1 mM Mg^2+^, GG + 1000 µg/mL acai aqueous extract (**B**); GG, GG + 0.001 µg/mL acai aqueous extract, GG + 1 µg/mL acai aqueous extract (**C**). Arrows indicate the point at which the agents were applied. Histograms are presented as the means of recordings of percentage normalized currents ± SEM for 20 cells (n = 20) (**D**). Differences between means were evaluated using a one-way ANOVA with Dunnett’s multiples comparisons post-test. For marked significance: *** *p* < 0.001 and **** *p* < 0.0001 when compared to a control cell exposure to 300 μM L-Glu + 10 μM Gly.

The expression and functional activity of ionotropic glutamate receptors (iGluRs) were examined using whole-cell patch clamping. Patch clamp recordings were taken from differentiated TE671 cells in the whole cell configuration at a holding potential of −50 mV and using a magnesium (Mg^2+^)-free perfusion solution. There were significant inward currents observed following the administration of 300 µM L-Glu + 10 µM Gly (Figure 6A), which could be reduced by the application of the NMDAR antagonists, 1 µM MK-801 and 1 mM Mg^2+^, which attenuated the response by approximately 65% and 45%, respectively (Figure 6A–C). Exposure of the differentiated TE671 cells to the acai berry aqueous extract at concentrations of 0.001, 1, and 1000 µg/mL triggered a significant inhibition (32%, 49%, and 50%, respectively) of the 300 µM L-Glu + 10 µM Gly-activated currents (Figure 6B–D).

### 3.7. Phytochemicals from Acai Berry Extracts Display High Affinity Binding to the NMDAR by Molecular Docking

Molecular docking analysis of the compounds from acai berry extracts [27] was performed using the Schrödinger suite, version 13.2, LLC, 2022.2 (New York, NY, USA). The identified compounds were docked in the groove of the primary agonist binding site in the GluN2B subunit, within the GluN1/GluN2A ligand binding domain (LBD). Glide was used to predict ligand binding affinities and rank them according to their Glide score as an estimate of relative binding affinities. The tabulated data for the binding parameters of the top 5 hit compounds with the strongest binding affinities out of the screened library are shown in Table 1. The docking scores of the top hits ranged from −10.041 kcal/mol (for the reference compound, glutamate) to −6.933 kcal/mol for histidine. The remaining ligands were arginine, 2,5-dihydroxybenzoic acid, threonine, and protocatechuic acid, with binding affinities of −8.423, −8.288, −7.320, and −7.103 kcal/mol, respectively.

**Figure 7:**
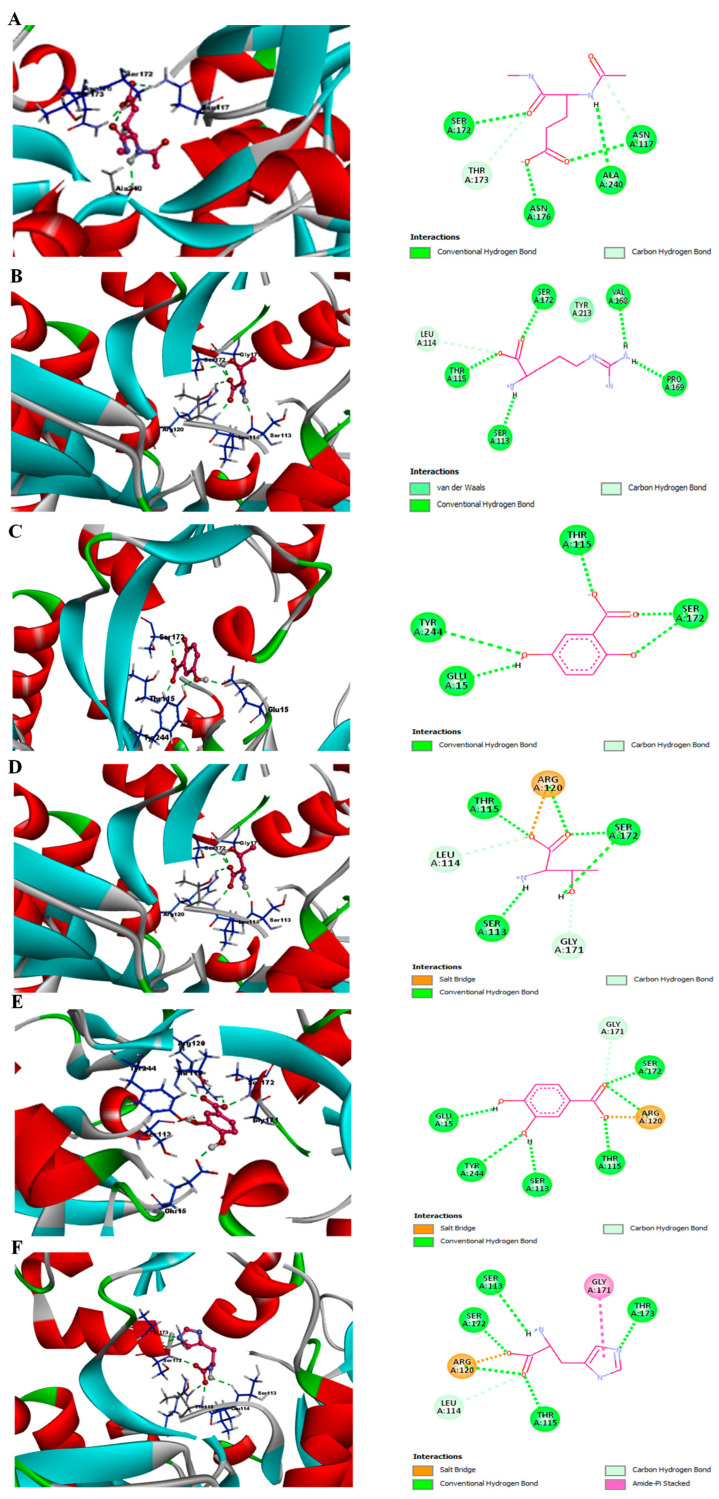
Predicted binding conformations of the ligands within the GluN2A binding region of the NMDAR. In silico modeling of the 3D structures (**left panels**) and interacting residues (**right panels**) for (**A**) glutamate, (**B**) arginine, (**C**) 2,5-dihydroxybenzoic acid, (**D**) threonine, (**E**) protocatechuic acid, and (**F**) histidine, for binding to the NMDAR.

The structures of each of the hit compounds and reference ligand at the binding site of GluN2B are shown in Figure 7A–F. To study the specific binding pattern in detail, Biovia discovery studio analysis was also performed to visualize the hydrogen bonding that governs the molecular recognition and shape complementarity of each of the docked complexes.

Post-docking diagrammatic analysis of each selected ligand was performed using Maestro 13.0 and a Biovia discovery studio visualizer to provide interaction patterns between the ligands and the binding pocket-forming residues of the NMDAR. Interactions were divided into polar (or non-polar), and the (relatively strong) polar interactions have been included in Table 2.

The reference ligand, glutamate, forms 5 hydrogen bonds with residues Asn-117, Ser-172, Thr-173, Asn-176, and Ala-240 of GluN2A, as shown in Figure 7A. Collectively, these interactions result in a relatively high docking score of −10.041 kcal/mol. The next strongest interaction was between the target protein and arginine. The ligand docking score was −8.423 kcal/mol, with hydrogen bonds formed with Ser-113, Leu-114, Thr-115, Val-168, Pro-169, Ser-172, and Tyr-213 (Figure 7B). Arginine may also form some π–π interactions within the NMDA receptor binding pocket due to its planar guanidinium group. This can stack with aromatic rings or interact with the π-electron systems of nearby residues or ligands, facilitating the stabilization of the ligand–protein complex and contribute to the relatively high docking score. The compound 2,5-dihydroxybenzoic acid displays relatively high binding affinity with a docking score of −8.288 kcal/mol and forms hydrogen bonds with Glu-15, Thr-115, Ser-172, and Tyr-244 (Figure 7C). Threonine has a binding energy of −7.320 kcal/mol attributed to the cumulative strong binding from conventional hydrogen bonds formed with Ser-113, Leu-114, Thr-115, Arg-120, Gly-171, and Ser-172 (Figure 7D). Protocatechuic acid has a docking score of −7.103 kcal/mol and forms seven hydrogen bonds with residues: Glu-15, Ser-113, Thr-115, Arg-120, Gly-171, Ser-172, and Tyr-244 (Figure 7E). Histidine displays the lowest theoretical docking score of −6.933 kcal/mol and forms conventional hydrogen bonds with Ser-113, Leu-114, Thr-115, Arg-120, Gly-171, Ser-172, and Thr-173 (Figure 7F).

### 3.8. MM/GBSA Studies

The docked ligands were further evaluated by calculating the binding free energy (∆Gbind) of protein–ligand complexes using Prime MM/GBSA studies [41,42]. The ∆Gbind values of all selected ligands were between −11.252 kcal/mol (2,5-dihydroxybenzoic acid) and −42.676 kcal/mol (threonine) (refer to Table 1), for which the more negative value signifies a stronger binding. Hence, the binding of histidine, arginine, and threonine was superior to L-glutamate, which was used as the reference compound.

#### Molecular Dynamics Simulation

**Figure 8:**
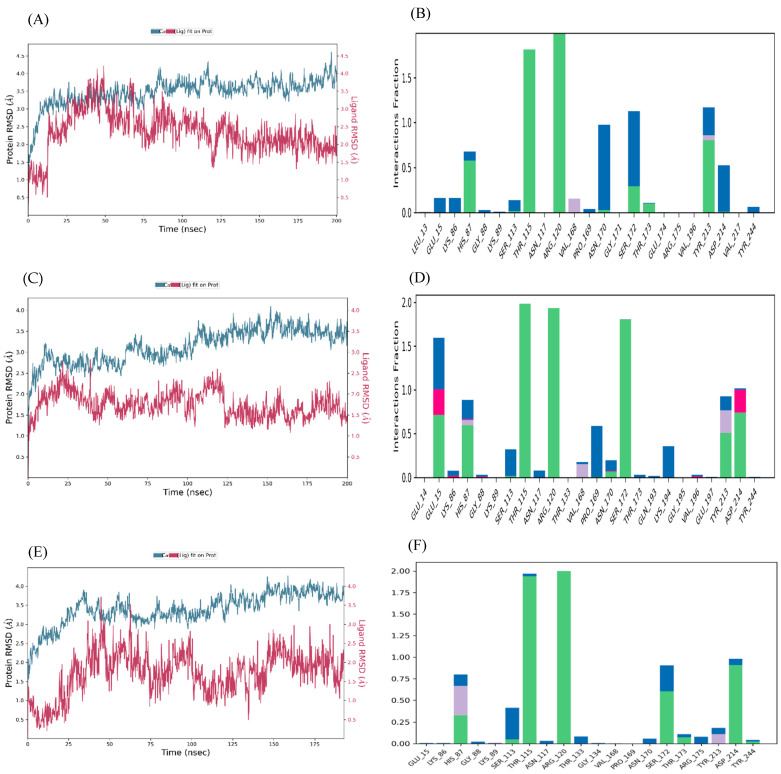
MD simulation studies of ligands in complex with the NMDAR. (**A**) RMSD plot of protein backbone (Cα) (blue color) and protein conformational change during glutamate binding. (**B**) Interaction fraction plot showing different NMDAR residues that interact with glutamate during a 200 ns MD simulation. (**C**) RMSD plot of protein backbone (Cα) (blue color) and protein conformational change during arginine binding. (**D**) Interaction fraction plot showing different NMDAR residues that interact with arginine during a 200 ns MD simulation. (**E**) RMSD plot of protein backbone (Cα) (blue color) and protein conformational change during 2,5-dihydroxybenzoic acid binding. (**F**) Interaction fraction plot showing different NMDAR residues that interact with 2,5-dihydroxybenzoic acid during a 200 ns MD simulation. For interactive plots: water bridges (blue), H-bonds (green), hydrophobic (purple), ionic (cerise).

The ligand–NMDAR (GluN2A) complexes with the three highest docking scores (glutamate–NMDAR, arginine–NMDAR, and 2,5-dihydroxybenzoic acid–NMDAR) were subjected to molecular dynamics (MD) simulations at NPT for 200 ns. The binding stability of the heterodimer-NMDA complexes were considered using root mean square deviation (RMSD) fingerprints and protein–ligand (P–L) interaction mapping. The RMSD plots measured the conformational changes in the NMDA backbone when bound to the respective ligand in comparison to its initial spatial conformation, and intermolecular contacts of NMDA and the bound ligands were recorded as interaction fraction mapping. Glutamate forms a stable complex with the NMDAR (Figure 8A,B), as does arginine (Figure 8C,D) and 2,5-dihydroxybenzoic acid (Figure 8E,F).

The RMSD plot for glutamate and the NMDAR indicates that the complex displays optimum stability with inherent fluctuations between 5 and 30 ns, and 96, and then onward until 200 ns (Figure 8A). From the interaction fraction plot of the bound complex, Arg-120 forms 100% stable hydrogen bonds and water bridges. Thr-115 and Tyr-213 form 95% and 40% stable hydrogen bonds and water bridges. Furthermore, Ser-172, Thr-173, and His-87 also contribute to stabilizing the protein–ligand complex during 5-30% of the simulation time (Figure 8B). The RMSD plot for arginine and NMDAR indicates that the complex is stabilized between 17 and 28 ns and at 36-37 ns (Figure 8C). The interaction plot of NMDA with arginine indicates that Thr-115, Arg-120, and Ser-172 form hydrogen bonds and water bridges for 95, 94, and 88%, respectively, of the simulation time. Furthermore, Glu-15, His-87, Tyr-213, and Asp-214 are some of the other protein residues which contribute to stabilizing the protein–ligand complex during 30-40% of the simulation time (Figure 8D). An initial MD simulation of the 2,5-dihydroxybenzoic acid-NMDAR complex for 200 ns did not show substantial configurational complementarity but eventually stabilized between 40 and 45 ns and 68 and 70 ns (Figure 8E). The interaction plot of NMDA with 2,5-dihydroxybenzoic acid indicates that Arg-120 and Thr-115 form hydrogen bonds and water bridges for 95% and 94% of the simulation time. In addition, Asp-214 forms hydrogen bonds, water bridges with the ligand for 40% of the simulation time, and Ser-172 and His-87 forms hydrogen bonds and water bridges for 30% and 15% of the simulation time (Figure 8F).

## 4. Discussion

Post-stroke cerebral damage arises from oxidative stress and excitotoxicity, and for the latter, is mediated by the excessive activation of L-Glu responsive NMDARs [13,14,15,16]. Hence, the aim of our study was to consider the molecular mechanisms associated with L-Glu neurotoxicity and whether the extracts from the acai berry are neuroprotective. Our results show that L-Glu induced cell cytotoxicity in differentiated TE671 cells and decreased cell viability in a concentration-dependent manner. Whereas, acai berry extracts were only slightly neurotoxic themselves at high concentrations and could reduce the L-Glu neurotoxicity. This was evidenced by less L-Glu-induced cell death and an amelioration of the L-Glu-induced depletion of cellular ATP, the MMP, and the induction of ROS and associated redox stress. Additionally, acai berry extracts attenuated the L-Glu activation of iGluRs. Virtual screening of the compounds present in acai berry extracts identified several molecules that could substitute for L-Glu binding to the NMDAR, and thereby provide a natural and potentially beneficial, competitive NMDAR antagonist.

### 4.1. Viability of Cells in Response to L-Glu and Acai Berry Extract

The L-Glu-induced cell cytotoxicity and reduced viability was concentration-dependent (Figure 1A), with approximate IC_50_ values of 88 and 67 mM after 24 and 48 h exposures, respectively, for MTT assays. These values are comparable to those for L-Glu cytotoxicity to human neuroblastoma SH-SY5Y cells [27,43,44]. Previous studies have shown that L-Glu can induce neuronal death over a concentration range of 0.1 to 100 mM [27,43,44]. The cytotoxicity of L-Glu to TE671 cells was further validated using LDH assays, as an independent method for the determination of cell viability [35,45]. LDH assays confirmed the L-Glu concentration-dependent reduction in cell viability (Figure 1C). Initially, cell metabolic activity (as measured via an MTT assay) was increased in the differentiated TE671 cells after treatment with acai berry extracts alone at concentrations of 0.001 to 1000 µg/mL for 24 h, and this is in keeping with other studies that have suggested that certain phytochemical extracts can stimulate cell proliferation [27,46,47]. However, relatively high concentrations of acai berry aqueous extract (10 to 1000 µg/mL) induced a reduction in cell viability, as observed in other studies [48,49,50,51]. An examination of the ability of acai berry extracts to reduce L-Glu toxicity demonstrated their neuroprotective potential via ameliorating the L-Glu-induced reduction in cell viability (Figure 2A–E), consistent with similar studies using SH-SY5Y cells [27].

### 4.2. Impact of L-Glu and Acai Berry Extracts on Mitochondrial Function

In line with other published studies, L-Glu exposure resulted in cellular bioenergy dyshomeostasis by reducing ATP production (Figure 3A,B) [27,52,53,54,55,56]. The lower concentrations of the acai berry extracts provided neuroprotective effects and ameliorated the L-Glu reduction in ATP levels, whereas the higher extract concentrations induced additional toxicity and a further lowering of cellular ATP levels (Figure 3C–F), an observation previously made with SHSY-5Y cells [27].

Lowered MMP values were recorded after L-Glu treatments for 24 or 48 h indicative of mitochondrial damage. Other in vitro studies support these findings that L-Glu impairs the MMP and mitochondrial functionality [27,57,58,59]. Similar to their effects on total cellular ATP levels, relatively low acai berry extract concentrations (0.001 to 10 µg/mL) were mitoprotective and capable of restoring some of the diminished MMP triggered by L-Glu exposure (Figure 4C,D).

L-Glu-induced mitochondrial damage was correlated with the liberation of ROS. The highly reactive nature of ROS can result in cellular damage via the oxidation of DNA, proteins, and lipids, and this can trigger mitochondrial damage and neuronal death [60,61,62]. L-Glu induced a concentration-dependent increase in ROS levels after 3 or 6 h in differentiated TE671 cells, which is consistent with prior research showing L-Glu-induced oxytosis [27,43,55,56,63,64]. Generally, higher acai berry extract concentrations induced ROS production but the relatively low concentrations of both aqueous and ethanolic extracts were able to reduce the total levels of ROS induced by L-Glu, consistent with potent antioxidant activity [24,27,33,46,47,49,51]. The acai berry extracts could also contain compounds able to induce and/or activate endogenous antioxidant defense, but we have not yet investigated this further.

In contrast to our previous studies with undifferentiated and differentiated SH-SY5Y cells, there was a responsive current to L-Glu + Gly stimulation in the dbcAMP-differentiated TE671 cells (Figure 6A). The L-Glu + Gly-activated currents could be inhibited by the NMDAR antagonists MK-801 and Mg^2+^ (Figure 6A,C). Previous research has shown that 1 µM MK-801 could completely block NMDAR activity [65], implying that the activated current in our study could arise predominantly from NMDAR-mediated activity but also with approximately one-third from a non-NMDAR mediated source. Certainly, this research confirms the activity of iGluR in differentiated TE671 cells, in keeping with the report that an α-amino-3-hydroxy-5-methyl-4-isoxazolepropionic acid receptor (AMPAR) agonist evoked a current response in undifferentiated TE671 cells and with evidence of the expression of NMDAR, AMPAR, kainate receptor (KAR), and metabotropic glutamate receptor (mGluR) subunits [30]. Likewise, the expression of iGluR subunits: GluA4, GluN1, GluK5, and GluN2D has been reported in TE671 cells [29], as was the expression of GluN1 and the GluA2/3 subunits of the AMPAR [31]. Therefore, our study provides experimental evidence of iGluRs’ activity in TE671 cells, and it is therefore assumed that the L-Glu toxicity arises, at least in part, via L-Glu over-stimulation (excitotoxity)of iGluRs.

In addition, the current study confirmed that the acai berry aqueous extract inhibited the L-Glu + Gly-activated currents in differentiated TE671 cells (Figure 6B,C). Acai berries contain a range of pharmacologically active substances, one or more of which may be responsible for this inhibition [27,66,67]. There are a number of plant extracts and herbal products that can inhibit the glutamatergic excitation mediated by the NMDAR [67,68,69,70]. Furthermore, a diverse group of naturally occurring compounds, including flavonoids, alkaloids, terpenoids, and fatty acids can bind directly to the NMDAR and act as antagonists [67,68,69,70]. According to our previous liquid chromatography–mass spectrometry (LC-MS) results [27], acai berry extracts retain several phytochemicals with established antioxidant [33,71,72,73,74,75,76,77,78] and neuroprotective properties [79,80,81]. Any of these phytochemicals could provide neuroprotection against the L-Glu excitotoxicity. NMDAR antagonist activity and prevention of L-Glu excitotoxicity have been reported for some acai berry phytochemicals using in silico or in vitro analyses; such as chlorogenic acid [82], quercetin, gallic acid, protocatechuic acid, and vanillic acid [83]. We virtually screened all the compounds detected in these acai berry extracts [27], and this resulted in the identification of five compounds with relatively strong and potentially stable binding to the LBD of the NMDAR (Table 1 and Figure 7 and Figure 8). This included protocatechuic acid, supporting previous in silico studies [83]; a number of amino acids with, as yet, unrecognized and potentially novel NMDAR antagonism properties; and 2,5-dihydroxybenzoic acid, which is also not a recognized NMDAR antagonist, but interestingly, 2-hydroxy-5-(2,3,5,6-tetrafluoro-4-trifluoromethyl-benzylamino)-benzoic acid, which shares a 2,5-dihydroxybenzoic acid structure, reversibly suppressed NMDAR responses (as an uncompetitive inhibitor), evaluated using whole-cell patch clamping [84].

A limitation of our study is that we have yet to determine unequivocally which phytochemical(s) ameliorate the L-Glu activation of the NMDAR. Furthermore, activity may not be entirely directed at NMDARs, since dTE671 cells also express non-NMDAR type iGluRs, albeit at lower levels than the NMDAR (Figure 6 and Figure S3). In order to assess and validate the antagonistic activity of phytochemicals at the LBD of the NMDAR, individual electrophysiological measurements will be required, and this is beyond the scope of this current manuscript but will be considered in future studies. It is also possible that phytochemicals could bind to other regions of the NMDAR to affect NMDAR activity. However, typical ligand screening is focused on the LBD and uses the GluN1/GluN2B LBD dimer in complex with glutamate and glycine analogs for the virtual screening and docking of orthosteric ligands, due to the high-resolution crystal structure and stable conformation of the LBD dimer.

Strategies that utilize the competitive or uncompetitive inhibitors (antagonists) of NMDARs may prove useful for the treatment of post-ischemic stroke [14,15,16], and phytochemicals may represent novel chemical entities with this mode of action. However, a drawback to this therapeutic approach is that signaling networks downstream of the target receptors could be activated post-stroke; hence, they will have already triggered cellular damage and neuronal loss. Thus, in the absence of timely intervention by agents capable of attenuating excitotoxicity and oxidative stress, there is a need to consider therapies that target specific NMDAR subtypes and those that block downstream death-related pathways [85,86] and/or neuroprotective agents that could be administered prophylactically.

## 5. Conclusions

**Figure 9:**
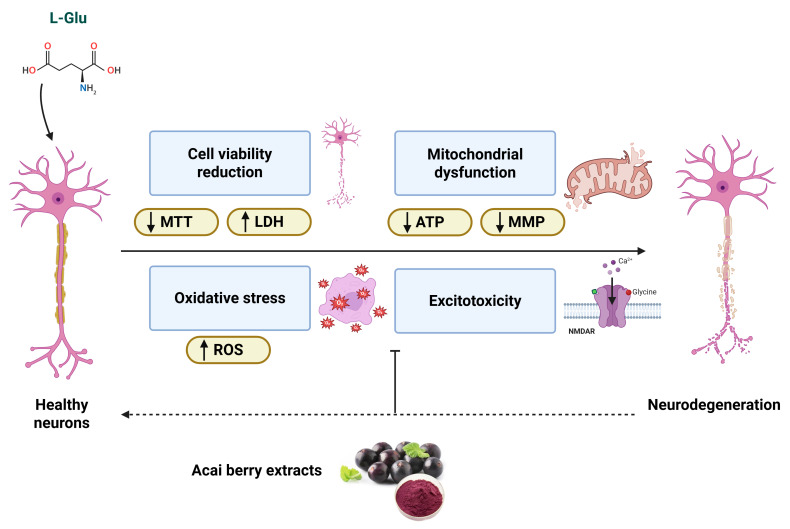
A summary of the neuroprotective properties of the acai berry.

Despite the low-level cytotoxicity that arises from relatively high acai berry extract concentrations, the co-application of acai berry extracts with L-Glu provided neuroprotection by limiting cell viability loss, restoration of the depleted mitochondrial function, and lowering oxidative stress and excitotoxicity—as summarized in Figure 9. Thus, acai berry extracts may contain potentially useful biologically active compounds that are neuroprotective against the neuropathology mediated by oxidative stress and excitotoxic mechanisms, such as those encountered in strokes. To date, dietary supplementation with acai berries has been utilized in human clinical trials that have focused on improved metabolic health [87,88,89], but human studies have not yet considered the benefits of acai berries in neuroprotection or stroke treatment. It is therefore appreciated that this is a preliminary study and one that only considers plant extracts as a starting point for the detection of phytochemicals with useful biological properties. Further studies are required that will include compound isolation and subsequent in vitro and in vivo assessment in appropriate animal models before any evaluation of their beneficial neuroprotective application for combatting strokes in humans can be undertaken.

L-Glu can induce neurotoxicity and reduce neuronal viability measured via MTT and LDH assays. L-Glu neurotoxicity is mediated by excitotoxicity via NMDAR activation, mitochondria damage with reduced mitochondrial membrane potential (MMP) and ATP production, and the generation of reactive oxygen species (ROS) that contribute to cellular redox stress. Acai berry extracts sustain cell viability, attenuate the NMDAR activation and excitotoxicity, restore mitochondrial dysfunction, and limit the induction of oxidative stress.

## Figures and Tables

**Table 1:** Molecular docking and Prime MM/GBSA result for ligands screened against the NMDAR target protein.

						Prime MM/GBSA
Ligand	Molecular Formula	Molecular Weight	Docking Score	XPG Score	Glide Score	Δ Gbind
		g/mol	kcal/mol	kcal/mol	kcal/mol	kcal/mol
Glutamate	C_8_H_13_N_2_O_4_	201.20	−10.041	−10.041	−10.041	−15.897
Arginine	C_6_H_15_N_4_O_2_	175.21	−8.423	−8.423	−8.423	−40.208
2,5-Dihydroxybenzoic acid	C_7_H_5_O_4_	153.12	−8.288	−8.289	−8.289	−11.252
Threonine	C_4_H_9_NO_3_	119.12	−7.320	−7.320	−7.320	−42.676
Protocatechuic acid	C_7_H_5_O4	153.12	−7.103	−7.103	−7.103	−14.978
Histidine	C_6_H_9_N_3_O_2_	155.15	−6.933	−7.394	−7.394	−27.708

**Table 2:** Polar H-bond interacting residues with ligands in GluN2A of the NMDAR.

	Interaction Type	Interacting Residues
Glutamate	Polar	Asn-117, Ser-172, Thr-173, Asn-176, Ala-240
Arginine	Polar	Ser-113, Leu-114, Thr-115, Val-168, Pro-169, Ser-172, Tyr-213
2,5-Dihydroxybenzoic acid	Polar	Glu-15, Thr-115, Ser-172, Tyr-244
Threonine	Polar	Ser-113, Leu-114, Thr-115, Arg-120, Gly-171, Ser-172
Protocatechuic acid	Polar	Glu-15, Ser-113, Thr-115, Arg-120, Gly-171, Ser-172, Tyr-244
Histidine	Polar	Ser-113, Leu-114, Thr-115, Arg-120, Gly-171, Ser-172, Thr-173

## Data Availability

The results’ data associated with this manuscript are included as Supporting Data.

## Supplementary Materials

The following supporting information can be downloaded at: https://www.mdpi.com/article/10.3390/brainsci15101073/s1, Figure S1: Morphological characteristics of undifferentiated and differentiated TE671 cells.; Figure S2: X ray crystal structure of the apo human GluN1/GluN2A LBD (NMDA with PDB ID: 5H8F). Figure S3: Effects of iGluR agonists, non-NMDAR agonists and a NMDAR-selective antagonist on dTE671 cells using whole-cell patch clamp recordings. Table S1: Compounds detected in acai berry extracts detected by LC-MS.
