# Supplementary material for: Acai Berry Extracts Can Mitigate the L-Glutamate-Induced Neurotoxicity Mediated by N-Methyl-D-Aspartate Receptors
Source: Brain Sci. 2025 Oct 1;15(10):1073. doi: 10.3390/brainsci15101073 (PMC12564672; doi:10.3390/brainsci15101073)
Supplement: Supplementary file 1 [file brainsci-15-01073-s001.zip › brainsci-3815298-supplementary.pdf]

Supplementary Figure S1. Morphological characteristics of undifferentiated and differentiated TE671 cells.

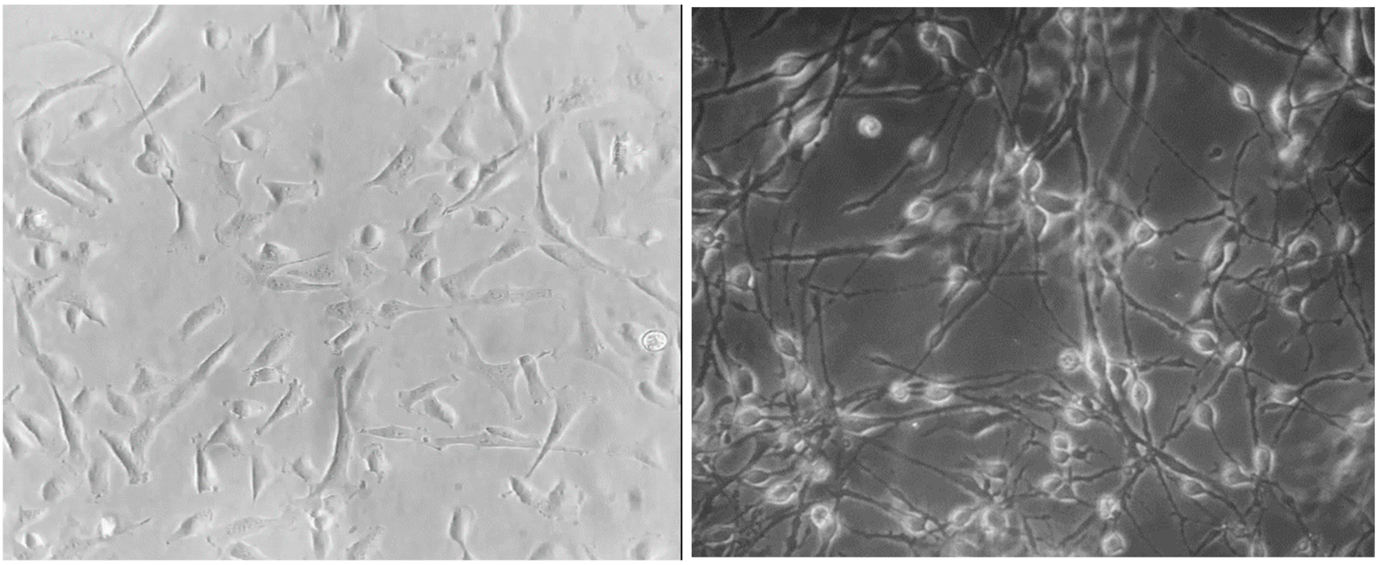

Undifferentiated TE671 cells Differentiated TE671 cells

Undifferentiated TE671 cells are round and elongated cells with spindle-shaped extensions. After differentiation with N6,2′-O-dibutyrylladenosine 3′,5′-cyclic monophosphate (dbcAMP), the differentiated TE671 cells showed extended neurite arborization. Images were captured with phase contrast light microscopy at x400 magnification.

Supplementary Figure S2. X ray crystal structure of the apo human GluN1/GluN2A LBD (NMDA with PDB ID: 5H8F).

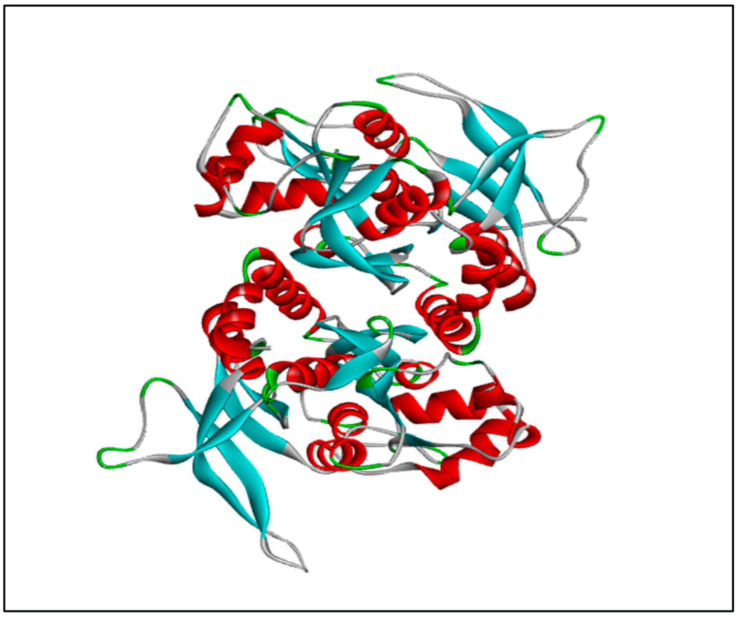

Supplementary Figure S3. Effects of iGluR agonists, non-NMDAR agonists and a NMDAR-selective antagonist on dTE671 cells using whole-cell patch clamp recordings.

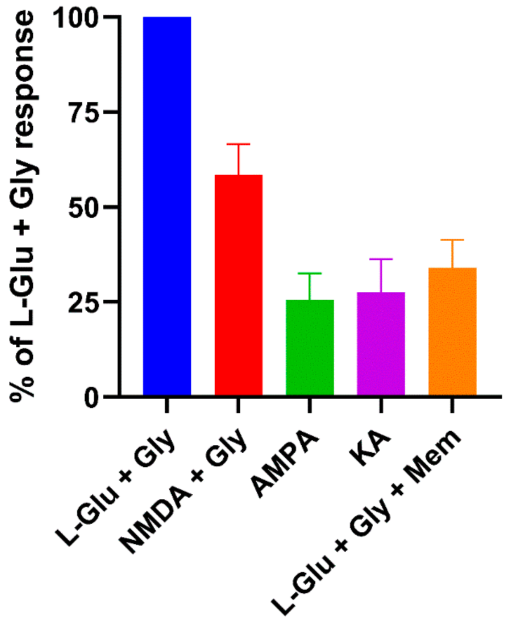

Relative size of dTE671 cell whole-cell currents, normalized to the response to 100 µM L-Glu + 100 µM Gly for: 100 µM NMDA + 100 µM Gly; the non-NMDAR agonists 100 µM AMPA and 100 µM kainic acid (KA); and 100 µM L-Glu + 100 µM Gly + 100 µM memantine (Mem), an NMDAR-selective antagonist.

Memantine at this concentration (100 µM)should inhibit the majority of the NMDAR mediated L-Glu + Gly elicited current. Bars are mean normalized (%) current ± SEM, n = 16 cells.

Supplementary Table S1. Compounds detected in acai berry extracts detected by LC-MS.

Name of compound	Compound class
Protocatechuic acid	**Phenolic compounds and phenolic acids**
Syringic acid	
Vanillic acid	
Gallic acid	
4-Hydroxybenzoic acid	
Benzoic acid	
2,5-Dihydroxybenzoic acid	
Chlorogenic acid	
Dihydrokaempferol	**Flavonoids**
Luteolin	
Quercetin	
Taxifolin deoxyhexose or Taxifolin	
Quercetin-3-O-rutinoside (rutin)	
Quercetin 3-O-glucoside (Isoquercitrin)	
Kaempferol rhamnoside	
Isoorientin	
(+)-isolariciresinol	**Lignans**
(+)-lariciresinol	
Dihydroconiferyl alcohol	
Catechin (+)	**Proanthocyanidin**
(+)-menthiafolic acid	**Monoterpenoids**
(E,Z)-2,6-dimethyl-2,6-octadiene-1,8-diol	
(E,E)-2,6-dimethyl-2,6-octadiene-1,8-diol	
(-)-loliolide	**Norisoprenoid**
**Major fatty acids**	
Oleic acid	**Monounsaturated fatty acids**
Palmitoleic acid	
Linoleic acid	**Polyunsaturated fatty acids**
Linolenic acid	
Palmitic acid	**Saturated fatty acids**
Stearic acid	
Alanine	**Amino acids**
Lysine	
Arginine	

Methionine	
Phenylalanine	
Proline	
Glutamic acid	
Serine	
Glycine	
Threonine	
Histidine	
Tryptophan	
Tyrosine	
Isoleucine	
Valine	
Leucine	
Cellotetraose	**Oligosaccharide (Tetrasaccharide)**
Sucrose	**Disaccharide**
Quinic acid isomer 1	**Hydroxy acids (Cyclitol derivative)**
Quinic acid isomer 2	

Compounds detected in acai berry aqueous and ethanolic extracts as published in [27]. Life 2023, 13, 1019, doi:10.3390/life13041019.
